# Wildfires as a Public Health Problem: a Setting for Nursing in Disasters

**DOI:** 10.17533/udea.iee.v37n3e01

**Published:** 2019-10-23

**Authors:** R. Mauricio Barría P.

**Affiliations:** 1 RN, M.Sc, DrPH. Director of the Institute of Nursing, Faculty of Medicine, Universidad Austral de Chile Chile. Member of the Center for Fire and Resilience Research of Socio-Ecological Systems. email: rbarria@uach.cl Universidad Austral de Chile Universidad Austral de Chile Chile rbarria@uach.cl

**Keywords:** air pollutants, wildfires, disasters, global warming, public health, pulmonary disease, chronic obstructive, professional role, health personnel.

Climate change and global warming are phenomena that are progressively affecting the planet. Temperature changes have been posing mayor threats for different domains of human society. One of these threats is related to health due extreme temperatures, and natural disasters. Thus, it is constituted as a global threat to biodiversity and the human population, since not only affects ecosystems equilibrium but increasing wildfires as consequence of increased temperatures and diminished precipitations. Moreover, wildfires have concentrated attention not only because of their ecological and environmental consequences but also for affecting production, economics, and health of people potentially exposed to environmental risks such fire, smoke, and others chemical products released from wood combustion process.

Wildfires have been steadily increased in recent decades, and are likely to continue according to the progress of climate change. Consequently, the impact of wildfire smoke on health will be increased in a near future.(1) Hence, it is to be expected that, knowing the increasing in frequency and intensity of extreme climate conditions, policies would be designed and implemented to prevent wildfires, along with public health strategies to coping smoke inhalation and to respond efficiently to their ravages.

Much of the understanding of the effects of wildfire smoke on health derives from studies on exposure to urban particles, principally fine particulate matter (PM_2.5_), derived from vehicular traffic and from other anthropogenic sources, like the use of fossil fuels for cooking and heating. There is already been reported an important burden to public health, in terms of frequency and economic cost of deaths and diseases related with these events.(2) Today, it is known that smoke from forest fires may increase dramatically the levels of PM_2.5_ with respect to periods when wildfires do not occur(3) and considering that PM_2.5_ originated from wildfires could have exceptionally high compositions or concentrations of specific chemicals that differ from that generated by other sources, it could also have a different response and outcomes on the exposed populations health. In this scenario, the knowledge gap on short- and long-term effects of exposure to wildfires will require multiple approaches and collaborative work to provide significant information on such exposure. For example, joint work among experts in monitoring air quality, wildfire smoke exposure modeling, toxicology, physiology, and epidemiology to seeking a better understanding in wildfires smoke impact on health.(4)

A review on the effects on health due to non-occupational exposure to wildfire smoke concluded that these fires have the potential of inducing an important burden to health, furthermore, proposing that because wildfires can occur with greater frequency and intensity due the effect of climate change, this burden may increase in the future.(3) Currently, sufficient evidence exists that the air contamination stemming from wildfires is associated consistently with respiratory effects, which can occur differently according to age and other sociodemographic factors.(5) A strong association exists between wildfires smoke exposure (PM_2.5_) and mortality due all causes, and respiratory morbidity. Specifically, association has been verified between this exposure and exacerbations of asthma and chronic obstructive pulmonary disease, bronchitis, and pneumonia.(6,7) Since the epidemiological data that linked exposure to smoke from wildfires with cardiovascular mortality and morbidity are mixed and inconclusive, further studies to establish its relation with cardiovascular morbidity and mortality in the population are needed.(3,6)

Although many countries have implemented policies and legislation to control environmental air contamination, including that in interior spaces, efforts to avoid and control wildfires and mitigate their consequences seem insufficient. Otherwise, key territorial factors envision that even if prevention policies exist, wildfires will continue occurring, hence, the focus should be on mitigating their effects. 

From a public health approach, besides recognizing the consequences of this environmental exposure, it is important to differentiate who within the population are the individuals potentially most affected and the environments where increased risks to health may exist. This is how it is recognized that smoke from wildfires can have a significant impact on subgroups of vulnerable population that are more sensitive even with exposure to lower concentrations; among them, children and adolescents, individuals with respiratory or cardiovascular compromise, the elderly, pregnant women, and cigarette smokers.(8) Also, an impact exists on the safety of the population in zones surrounding the fires in roadways and air traffic due to reduced visibility in zones affected by wildfires. Identifying vulnerable communities to wildfires smoke exposure effects allow to prepare responses, increase resilience, and improve public health outcomes during fire activity. In this regard, tools have been designed to establish the population vulnerability like the Community Health Vulnerability Index based on factors known for increasing the risks of effects on health due to air contamination and wildfires smoke exposure. Thus, it has been possible to differentiate most vulnerable zones and communities and identify those with the potential to benefit most from mitigation strategies to minimize exposure to smoke and diminish the economic and health burden imposed on the population.(9) Similarly, the Center for Disease Prevention and Control (CDC) develops an on-line tool that uses short-term predictions and prognoses of the concentrations of wildfires smoke to integrate them with measurements of vulnerability at population level to identify groups at risk. To develop this tool, contribution was sought from all stakeholders involved from academic, federal, state, local, tribal, and territorial settings.(10)

Currently, we are witnessing global-scale disasters, which require countries with a shared policy with planetary reach. At to date of this article, the catastrophe in the Amazon has revealed the ecological and environmental scope, as well as the effect to human health by megafires, like that commented. Besides the direct deterioration of the affected zone, death of different species and destruction of their habitat, smoke could affect strongly global climate, modifying rain patterns and terrestrial radiation balance. This emergency recently moved leaders from seven countries in the Amazon to undersign the Leticia Pact that seeks a cooperation network upon disasters, articulating national systems on disaster prevention and response.(11)

In turn, at local level, policies aimed at preserving ecosystems to fires must also consider public health, requiring an intersector and comprehensive approach. For this, in addition to considering resources to fight wildfires, we must include prevention, territorial planning, and community work strategies. 

As is other types of disasters, phases may be recognized in wildfires where nurses can act: mitigation, preparation, response, and recovery. Understanding these phases allow diminishing the effects of wildfires and increasing the community’s resilience. Mitigation includes activities of health promotion, professional and public education on health effects due to wildfires, and the preparation for public health needs. Preparation refers to activities nurses carry out to help individuals, families, and communities to understand what they must do to protect their health and stay safe during a wildfire. Nurse responses to disasters consider the type of response needed, their professional roles, if dealing with a local disaster or not, their own preparation, and personal obligations. Finally, recovery seeks to help communities to return to functional levels as fast as possible -resilience-.(12) 

In other scenario, have been proposed some interventions or actions that nurses must conduct from a clinical setting to avoid or minimize exposure to contamination during a wildfire and which look to: i) provide patients with knowledge and tools to avoid and/or manage wildfire smoke exposure; ii) minimize adverse effects of wildfire smoke exposure; iii) prepare and educate patients for future events of wildfire smoke.(8)

As it has been argued in this paper, wildfires have become more frequent and with greater severity. In response to this, planned and collaborative intersectoral interventions are required, among which are the actions of the health teams. The role of nursing in response to disasters has been evolved. Nowadays, training aimed to developing specialists and leaders to response efficiently and successfully these events are required, focusing care on vulnerable groups and communites during disaster response.
